# Spatial Patterns and Risk Assessment of Heavy Metals in Soils in a Resource-Exhausted City, Northeast China

**DOI:** 10.1371/journal.pone.0137694

**Published:** 2015-09-28

**Authors:** Hongwei Chen, Jing An, Shuhe Wei, Jian Gu

**Affiliations:** 1 Key Laboratory of Pollution Ecology and Environmental Engineering, Institute of Applied Ecology, Chinese Academy of Sciences, Shenyang, China; 2 Institute of Applied Ecology, Chinese Academy of Sciences, Shenyang, China; 3 Shenyang University, Shengyang, China; Chinese Academy of Sciences, CHINA

## Abstract

Northeast China is an intensive area of resource-exhausted city, which is facing the challenges of industry conversion and sustainable development. In order to evaluate the soil environmental quality influenced by mining activities over decades, the concentration and spatial distribution of arsenic (As), cadmium (Cd), chromium (Cr), copper (Cu), nickel (Ni), lead (Pb), and Zinc (Zn) in surface soils (0-20cm) of a typical resource-exhausted city were investigated by analyzing 306 soil samples. The results showed that the average concentrations in the samples were 6.17 mg/kg for As, 0.19 mg/kg for Cd, 51.08 mg/kg for Cr, 23.27 mg/kg for Cu, 31.15 mg/kg for Ni, 22.17 mg/kg for Pb, and 54.21 mg/kg for Zn. Metals distribution maps produced by using the inverse distance weighted interpolation method and results revealed that all investigated metals showed distinct geographical patterns, and the concentrations were higher in urban and industrial areas than in farmland. Pearson correlation and principal component analysis showed that there were significant positive correlations (p<0.05) between all of the metals, and As, Cd, Cr, Mn, Ni, Pb, and Zn were closely associated with the first principal component (PC1), which explained 39.81% of the total variance. Cu and As were mainly associated with the second component (PC2). Based on the calculated Nemerow pollution index, percentage for slightly polluted (1<*P* ≤ 2) surface soils were reached 57.33%, while 42.65% topsoil samples are moderate polluted (2<*P*≤ 3). According to the results above-mentioned, different soil environmental function areas were classified and proper soil environmental management policy was proposed to decrease the environmental risks in the process of industrial city transformation.

## Introduction

Due to the non-renewable characters and cumulative effects of mineral resource, many mining cities are facing the crisis of resource exhaustion, and their development also face many difficulties[1]. In order to transit smoothly from industrial society to post-industrial society, and create a certain competitive and livable city, lots of resource-exhausted cities have transformed or are transforming. Therefore, the problem of how to fasten economy transformation and social development for the resource-exhausted mine city has been a hotspot nowadays.

Northeast China has rich mineral resources, fertile land, and a strong industrial base and has made great contribution to the construction of new China[2]. However, like the most resource-based cities, with the exploitation of resources and the gradual reduction of resource reserves, mining zones whose developments mainly depend on their own resources are facing downfalls[3]. In the meantime, abandoned mines, land subsidence, water pollution, hills of gangue and flyash by years of industrial and mining production in these cities cause the destruction and waste of resources, ecological degradation, and damage to the environmental landscape[4]. The transition of resource-based cities has become an irreversible trend in Northeast China. As the most important coal resource-exhausted city in northeast China, Fuxin city is the first pilot city of economic transition in China.

Fuxin is an important mineral-rich region in China. About 43 minerals are found in Fuxin, the dominant minerals extracted being coal, silica sand, zeolite, marble, and gold. Taking coal as an example, the cumulative amount of coal that has been produced in Fuxin has been estimated to be up to 600 million ton, and 790 million ton of coal reserves remain. Coal has been mined in Fuxin for more than 50 years, and these mining activities have produced 2667 ha of mining wasteland and 4,000 ha of spoil heaps. The development of coal resources brought great economic benefit, but caused a series of ecological and environmental problems such as mining subsidence and soil damage. According to the government policy and its development situation, Fuxin city has gradually stepped into the period of industrial transformation. Except mining industry, agriculture is the second pillar industry in Fuxin city. There are 7730km^2^ farmland in Fuxin, and 5.5 billion marketable grains are produced every year. With the course of the constantly industry transformation, modern agriculture has become the most important developing direction in Fuxin. Some land surrounding the mining area or urban area may be used as the agriculture soil. However, there is little research on the soil environmental quality in Fuxin.

As an important part of the environment, soil is very sensitive to environmental changes[5]. Long-term mining production and rapid urbanization have led to increasingly serious soil pollution problems in coal resource-exhausted city inevitably. The pollution of soils with heavy metals is the most notable of these problems [6], and it is directly related to food security and the human health [7, 8]. The contamination incident called “cadmium rice”, which occurred in China, illustrates the ecological risks posed by heavy metals, and these risks have become of global concern [9, 10]. Therefore, it is necessary to study the heavy metal pollution in soil and assess its potential ecological risk to the agricultural products and human health[11].

Heavy metal contamination can be assessed using various methods and criteria. The main assessment method that is used is the analysis of the spatial distributions of contaminants in a region [12], and this involves assessing the soil quality and locating the pollution sources[13] The complexities of and interactions within datasets have led to multivariate geostatistical and geographic information system methods being widely used to extract the most meaningful information from a dataset without losing useful information. Surveys of the concentrations of potentially toxic elements in soils provide the fundamental information required for environmental planning and management processes, so such surveys have been conducted in some regions in recent years [14,15]

The objectives of the research presented here were to (1) estimate the concentrations of heavy metals in surface soils in Fuxin, (2) determine the spatial distribution patterns of the heavy metal concentrations and the degree to which the soil is polluted with the heavy metals, and (3) assess the quality of the soil environment and potential risk, and provide appropriate soil environmental management measures for the cities in transition.

## Materials and Methods

### 2.1 Study area

**Fig 1:**
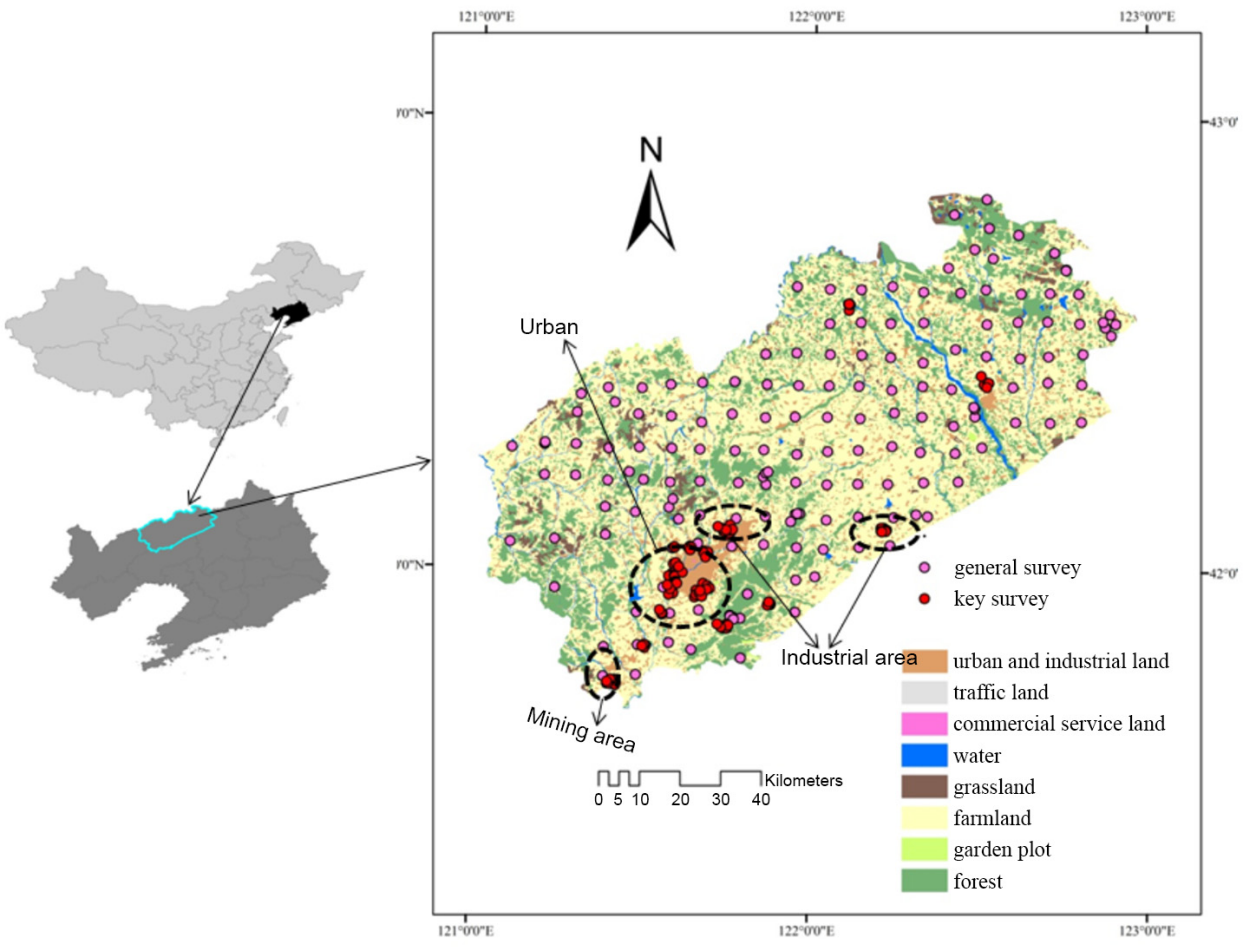
Location of Fuxin City in Liaoning Province, northeastern China and the sampling sites.

Fuxin is in an important mineral-rich region in Northeast China, and has a total area of 10355 km^2^, extending from 41° 41′ 19.52″ N to 42° 50′ 39.52″ N and from 121° 2′ 17.78″ E to 122° 58′ 33.81″ E (Fig 1). Fuxin is in the intergradational zone between the Inner Mongolian Plateau and the Liaohe River Plain, and it has a northern temperate continental monsoon climate. The main soil types, cinnamon soil is calcium carbonate’s leaching, illuviation and humification genesis with thin humus layer and middle or thick solum.

### 2.2 Sampling and chemical analysis

The authority responsible for the local government. No specific permissions were required for sampling the soil in the study area because the location is not privately-owned or protected in any way and these are no endangered or protected species in the soil sampled. A total of 306 soil samples were collected between October 2012 and May 2013 (Fig 1). A uniform grid method was used to determine the sampling sites in the general sampling area, using a regular 8 km × 8 km grid for arable land, a 16 km × 16 km grid for forested land, and a 40 km × 40 km grid for unused land. Moreover, the density of sample point was increased surrounding the urban area and industrial and mining enterprises. The locations of the sampling sites were determined using a hand-held global positioning system. Samples of soil from the soil surface to 15 cm deep were collected using a small shovel [16]. At each sampling site, soil samples (0–20 cm) were taken from five points, distributed as an ‘S’, and then the samples were fully mixed to form one sample. The samples were transferred to the laboratory, air-dried at room temperature for 10 d, and then sieved through a 2 mm mesh. The soil samples were then stored in glass bottles at 4°C until they were analyzed.

The heavy metals were extracted from the soil samples by microwave assisted acid digestion. A subsample of each soil was digested in 8 mL 65% nitric acid, 5 mL 30% hydrogen peroxide, and 3 mL 30% hydrofluoric acid[17], then the extract was evaporated to near dryness, and 10 mL Milli Q water was added. The extract was then stored in a 10 mL polyethylene vial at 4°C until it was analyzed. Triplicates were used for extracting each sample. The concentrations of the heavy metals were measured by ICP-OES (PE OPTIMA3000). The quality control measures that were taken were to 1) analyze 20 randomly selected samples and 9 national standard samples and 2) reanalyze a random selection of samples to ensure that the mean deviation between replicate analyses was less than 3%.

### 2.3 Data processing

Descriptive statistical parameters were calculated using the SPSS 19.0 software package. Pearson's correlation coefficient analysis and principal component analysis (PCA) were performed to identify relationships between the heavy metal concentrations in the soil samples and to identify the possible sources of the heavy metals [18].

### 2.4 Geostatistical methods

The spatial interpolation technique predicts values for cells in a raster from a limited number of sample data points. Interpolation is the process of predicting the values of an attribute at unsampled sites[19]. Unlike classic modeling approaches, spatial interpolation methods incorporate information about the geographic positions of the sample points [20]. The rationale behind spatial interpolation is that parameters for points that are close to each other are more likely than parameters for points further away to correlate and show similarities[21]. In the study presented here, the ordinary kriging method was used to determine the spatial distribution patterns for individual heavy metals and radial basis functions to express the Nemerow synthetic pollution index.

### 2.5 Pollution index method

The Nemerow pollution index was used to assess the quality of the soil environment across the sampling area. The Nemerow synthetic pollution index is widely applied to reflect the total pollution level and evaluate environmental quality. This method not only considers the average condition of the single-factor pollution index but also integrates its maximum. The heavy metal concentrations were examined to assess the soil pollution status against the Chinese Soil Quality Criterion GB 15618–2008 [22]. A single-factor pollution index was used to evaluate the presence of a specific metal and to assess the pollution levels in the sampling area. However, the Nemerow synthetic pollution index was used to reflect the overall pollution situation caused by the simultaneous presence of a number of metals [23]. The Nemerow index was calculated as shown below.

The single-factor pollution index was defined as *P*_*i*_ = *C*_*i*_ */ S*_*i*_, where *C*_*i*_ is the measured pollutant concentration and *S*_*i*_ is the natural background concentration of the pollutant.

The Nemerow synthetic pollution index was defined as *P*_N_ = { [(*Ci / Si*)_max_^2^ + (*Ci / Si*)_ave_^2^] / 2}, where *P*_N_ is the synthetic pollution index, (*Ci / Si*)_max_ is the maximum pollution index value for all of the pollutants, and (*Ci / Si*)_ave_ is the average pollution index value for all of the pollutants.

The *P* value indicates the degree of pollution, and the classifications used were: *P* ≤ 1, soil has not been contaminated; 1<*P* ≤ 2, soil has been slightly contaminated; 2 <*P* ≤ 3, soil has been moderately contaminated; and *P*>3, soil has been severely contaminated.

## Results and Discussion

### 3.1 Concentration and spatial distribution of heavy metals in soil

Descriptive statistics for the heavy metal concentrations in the soil samples analyzed in this study and background heavy metal concentrations found in Fuxin in 1990 are presented in Table 1. The heavy metal concentrations varied significantly in the soil samples. The heavy metal concentration ranges were 0.70–17.80 mg/kg for As, 0.004–0.94 for Cd, 9.87–129.49 for Cr, 5.5–62.29 for Cu, 0.34–83.10 for Ni, 4.15–63.63 for Pb, and 3.53–142.32 for Zn. Except for As and Cr, the mean concentrations of other metals were higher than the background concentrations for the heavy metals that were measured. This suggested that little external As and Cr was supplied to the soils in the sampling area. The concentrations of Cd, Cu, Ni, and Pb detected in this study were significant higher than the background concentrations. The great increases in the concentrations of these metals in soil that have occurred over the past 20 years indicate the extent of anthropogenic contributions of these metals to the environment in this area. The concentration of each metal varied significantly, and the coefficients of variation were large, ranging from 35.82% for Cu to 60.90% for As. These high coefficients of variation reflect the non-homogeneous distribution of anthropogenic heavy metals in the environment. The distributions of the heavy metals were skewed to lower concentrations, indicating that the concentrations of more samples were below than above the mean concentration. However, high heavy metal concentrations were found in samples from some of the sample sites in the urban, industrial and mining area, and these concentrations posed potential risks to environmental health.

**Table 1:** Concentrations of potentially toxic heavy metals in soil samples from Fuxin City.

Element	minimum	mean	median	maximum	SD	Variation coefficient	Skewness	Kurtosis	Background value ^a)^
	mg kg^-1^	mg kg^-1^	mg kg^-1^	mg kg^-1^		%			
As	0.70	6.17	4.05	17.80	3.76	60.90	0.80	-0.25	6.10
Cd	0.004	0.19	0.15	0.94	0.11	57.35	2.02	8.17	0.05
Pb	4.15	22.17	19.63	63.63	8.77	39.54	0.58	0.94	10.22
Cr	9.87	51.08	46.35	129.49	19.61	38.39	0.97	1.78	54.20
Cu	5.58	23.27	21.41	62.29	8.33	35.82	0.73	1.69	9.87
Zn	3.53	54.21	50.86	142.32	23.53	43.40	0.51	1.21	28.18
Ni	0.34	31.62	26.80	83.10	15.67	50.31	0.65	-0.03	14.57

^a)^ Cited from Wu, 1994

**Fig 2:**
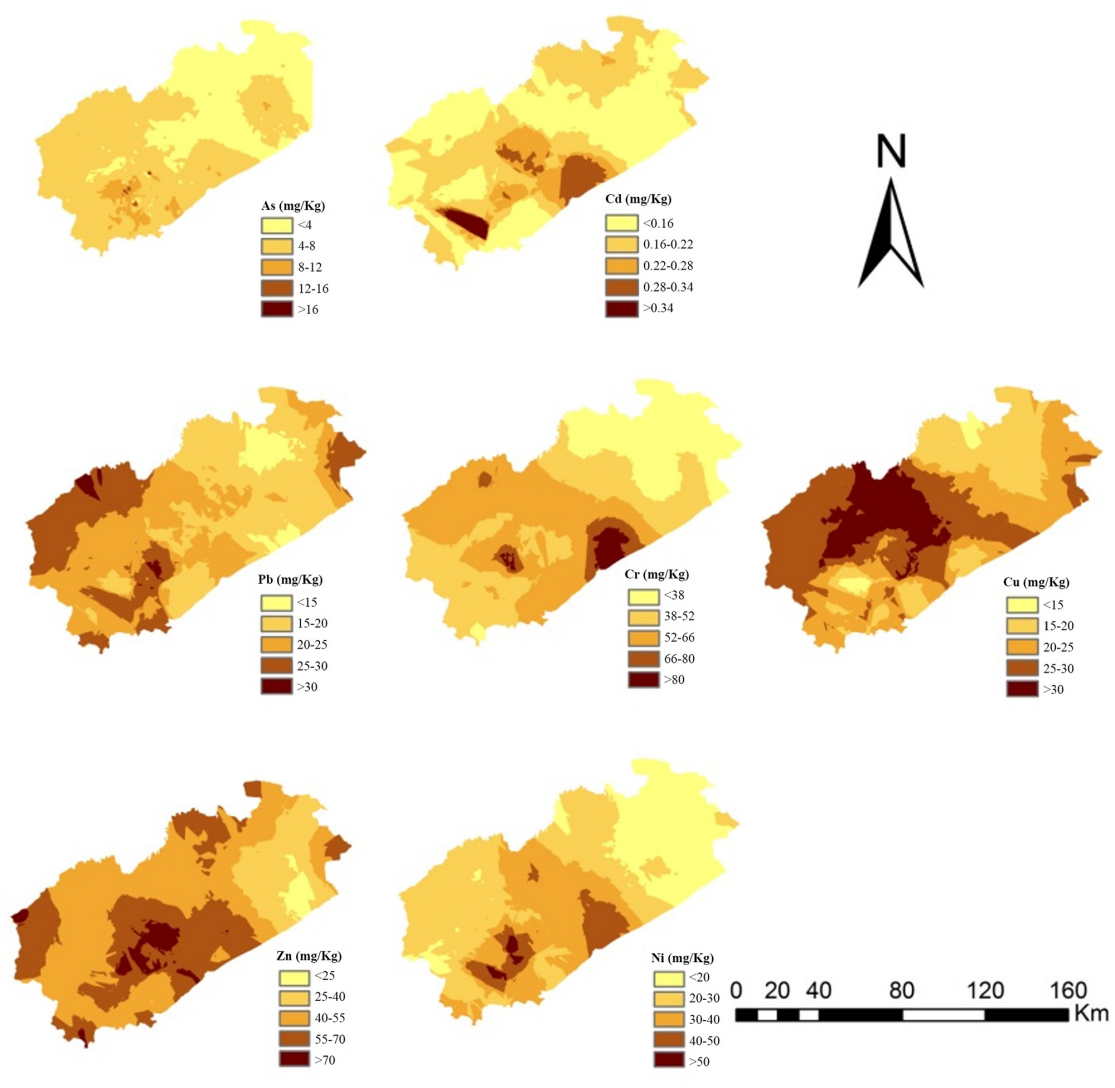
Spatial distributions of the heavy metals in soils in Fuxin city.

The spatial distribution of a metal is useful for assessing the possible sources of that metal in the sampling area and to identify hotspots where high metal concentrations are found. The geographical distributions of the heavy metals that were measured in the sampling area are shown in Fig 2. The highest concentration of Cd, Cr, and Ni was in the south central and southwestern parts of Fuxin where the urban area and large industry park was. The As concentrations were higher in the east than in the west of the study area, and extremely high As concentrations were only found at a small number of specific sites located in industrial and mining areas. The distribution of Pb and Zn was similar. Except the high concentration in the south central, the concentration of Pb and Zn were also high in the northwest region and a small region of east where is the main mineral area and agriculture area of Fuxin. The distribution of Cu was entirely different from the distributions of the other heavy metals. The Cu concentrations were high in the middle and northwestern part of Fuxin, and the highest Cu concentration was found at a copper smelting plant. The northwestern part of Fuxin is one of the major farming areas within the sampling area. It has been reported that one of the most important sources of Cu, Pb, and Zn to soil is agriculture, because of discharges of livestock manure, the use of chemical fertilizers and pesticides, and irrigation with wastewater[24]. For example, Cu and Zn are always present at background concentrations in livestock diets and may be added to certain feeds as supplementary trace elements for the health and welfare of the livestock or as growth promoters [25, 26]. The highest fraction of the amount of heavy metals consumed by livestock in feed is excreted in the feces or urine, and will therefore be found in the manure that is subsequently applied to the land. In this study, we found that the Cu, Pb, and Zn concentrations were notable high in the farming area of Fuxin. The concentrations of measured metals were relatively low in the northeastern part of Fuxin. The northeastern part of the city has a low population density and is mainly forested land and grassland. Variability in the large-scale distribution of a metal can usually be ascribed to different parent materials, soil types, and land use types[27]. However, considering the research scale of the study area, it suggest that the variability in the spatial distributions of the heavy metals was mainly caused by human activities such as urbanization, mining and agriculture.

The heavy metal concentrations in soils from areas dominated by different land uses are shown in Table 2. Statistical analysis suggested that the As, Cd, Ni, Pb, and Zn concentrations were significantly higher in soil samples from abandoned industrial land and urban and industrial land than from farmland and forested land. In contrast, the Cu concentrations were much higher in soil samples from farmland and forested land than other land use types. The Cr concentrations in soil samples from different land use types were not significantly different (p>0.05). The results indicated that human activity influenced the concentrations of the heavy metals that were measured. In particular, industrial activity increased the heavy metal concentrations in abandoned industrial land and urban and industrial land. We therefore suggest that environmental managers should take measures to decrease further soil pollution occurring in such areas.

**Table 2:** Heavy metal concentrations in soil samples from different land use types.

	As	Cd	Pb	Cr	Cu	Zn	Ni
	mg kg^-1^	mg kg^-1^	mg kg^-1^	mg kg^-1^	mg kg^-1^	mg kg^-1^	mg kg^-1^
Urban and industrial land	9.006*	0.207	23.360	51.997	17.897	55.933	36.974
Abandoned industrial land	7.451	0.214*	28.062*	51.570	22.211	63.446*	41.051*
Farm land	4.167	0.1722	20.299	49.681	26.232*	50.408	24.569
Forest land	5.009	0.1979	21.113	50.708	26.896*	53.966	31.328

### 3.2 Correlation and principle component analysis (PCA) on the heavy metals

Positive correlations between elements show that they have similarities of some sort [28]. The Pearson’s correlation coefficients and their significance levels (*P*<0.01) for the heavy metal concentrations in the soil samples are shown in Table 3. All of the heavy metal concentrations correlated positively with each other, and these correlations were statistically significant, which implies that the non-background concentrations of these heavy metals were supplied by similar pollution sources.

**Table 3:** Pearson correlation matrix for the heavy metal concentrations in the soil samples.

	As	Cd	Pb	Cr	Cu	Zn	Ni
As	1						
Cd	0.168**	1					
Pb	0.178**	0.181**	1				
Cr	0.259**	0.242**	0.283**	1			
Cu	-0.197**	0.159**	0.346**	0.356**	1		
Zn	0.147**	0.291**	0.384**	0.291**	0.311**	1	
Ni	0.288**	0.440**	0.411**	0.529**	0.181**	0.402**	1

The PCA method is a multivariate statistical analysis that can be used to identify the sources of contamination[18]. The PCA results for the metal concentrations found in the soils from Fuxin are shown in Table 4. After determining the initial eigenvalues, two principal components were considered, and these components accounted for more than 70.99% of the total variance. The communalities shown by the variables, considering the two components, varied from 0.335 for As to 0.778 for Ni. All of the elements were therefore represented well by the two principal components. The initial component matrix (Table 5) indicated that Cd, Cr, Ni, Pb, and Zn were closely associated with the first principal component (PC1), which explained 39.81% of the total variance, while Cu and As were mainly associated with the second component (PC2). Most of the heavy metals that were measured would have mainly been supplied by the industrial and mining activities, whereas Cu and As would mainly have been supplied by agricultural activities. This suggests that the concentrations of each heavy metal in the soil samples that were analyzed might have been affected by more than one source[29].

**Table 4:** Total variance explained by each component and the component matrices.

Component	Initial Eigenvalues			Extraction Sums of Squared Loadings		
	Total	% of Variance	Cumulative %	Total	% of Variance	Cumulative %
1	2.787	39.807	39.807	2.787	39.807	39.807
2	1.240	17.709	57.516	1.240	17.709	57.516
3	0.943	13.472	70.988			
4	0.747	10.668	81.656			
5	0.550	7.859	89.514			
6	0.411	5.873	95.388			
7	0.323	4.612	100.000			

**Table 5:** Principal component analysis loading matrix for the heavy metals.

	Cd	Pb	Cu	Zn	As	Ni	Cr
PC1	0.643	0.638	0.484	0.729	0.355	0.778	0.685
PC2	0.122	-0.164	-0.734	-0.095	0.779	0.207	0.020

### 3.3 Assessment of potential environmental risks

The Nemerow single-factor pollution indices for the 7 heavy metals that were measured are summarized in Table 6. The average *P*_*i*_ values for the heavy metals increased in the order of As < Zn < Cr <Pb< Cu < Ni < Cd. According to the *P*_*i*_ values, the soils in the sampling area are unpolluted with most of the heavy metals, except for Cd. However, among the 306 samples that were analyzed, 8 samples for As, 115 for Cd, 18 for Cr, 22 for Cu, 24 for Pb, 84 for Ni, and 9 for Zn contained concentrations higher than the Chinese standard for soil (GB15618-2008), indicating that some sample sites were polluted by these heavy metals.

**Table 6:** Single-factor pollution indices for the nine heavy metals.

	Minimum	Maximum	Mean	SD	Variance
As	0.05	1.19	0.41	0.25	0.06
Cd	0.02	2.92	1.01	0.57	0.32
Pb	0.12	1.82	0.63	0.25	0.06
Cr	0.11	1.25	0.56	0.21	0.04
Cu	0.16	2.82	0.68	0.30	0.09
Zn	0.04	1.42	0.54	0.24	0.06
Ni	0.01	3.15	0.86	0.55	0.30

**Fig 3:**
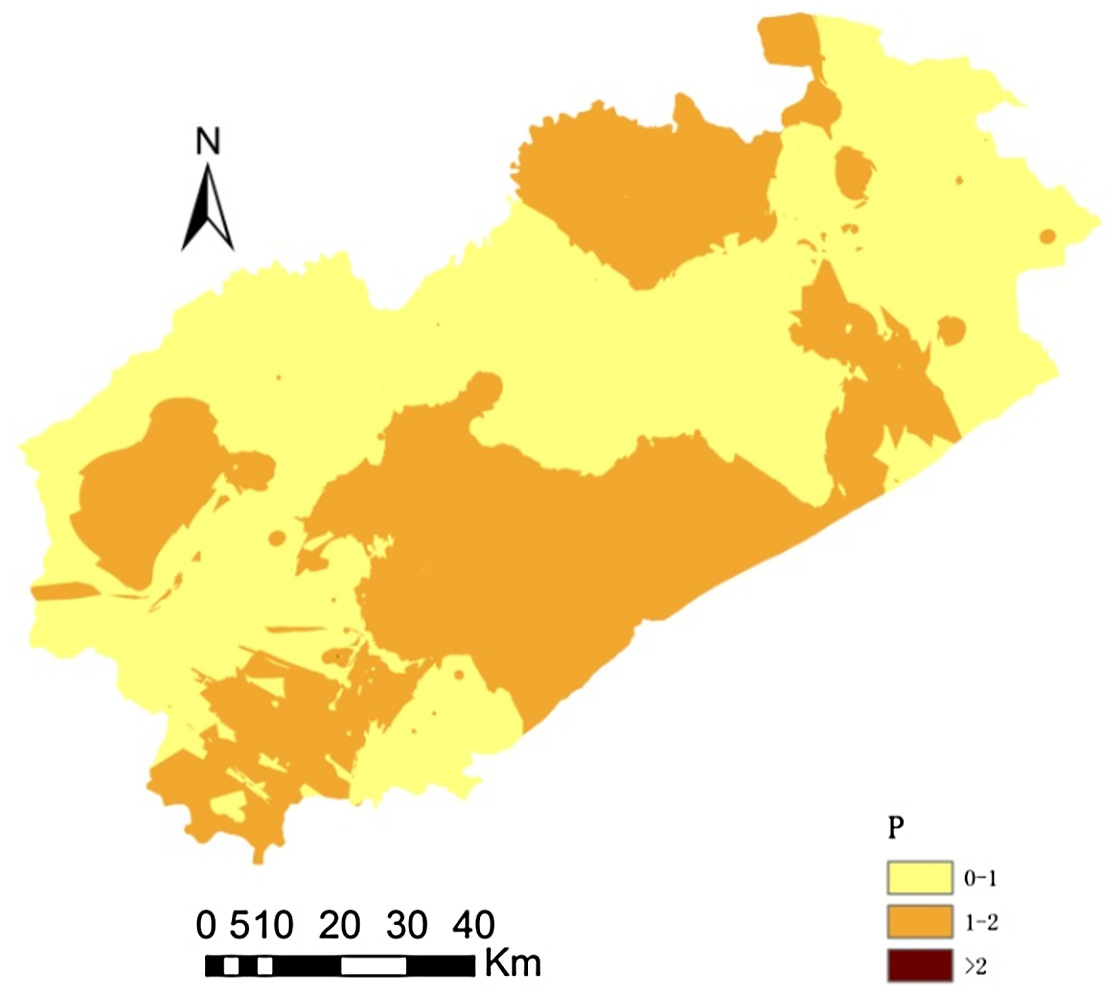
Pollution class distribution determined using the Nemerow synthetic pollution index.

Due to the complexity of soil, a synthetic pollution index can reflect the pollution level in soil better than a single-factor pollution index, which can only reveal the pollution level for one metal. We used the Nemerow synthetic pollution index and found that, of the 306 soil samples, 154 were safe/unpolluted, 146 were severely polluted, 6 were moderately polluted (these samples were from the area dominated by the chemical and industry). The areas affected by moderate pollution were mainly urban and mining areas (Fig 3).

### 3.4 Soil environmental function regionalization and pollution control measures

**Fig 4:**
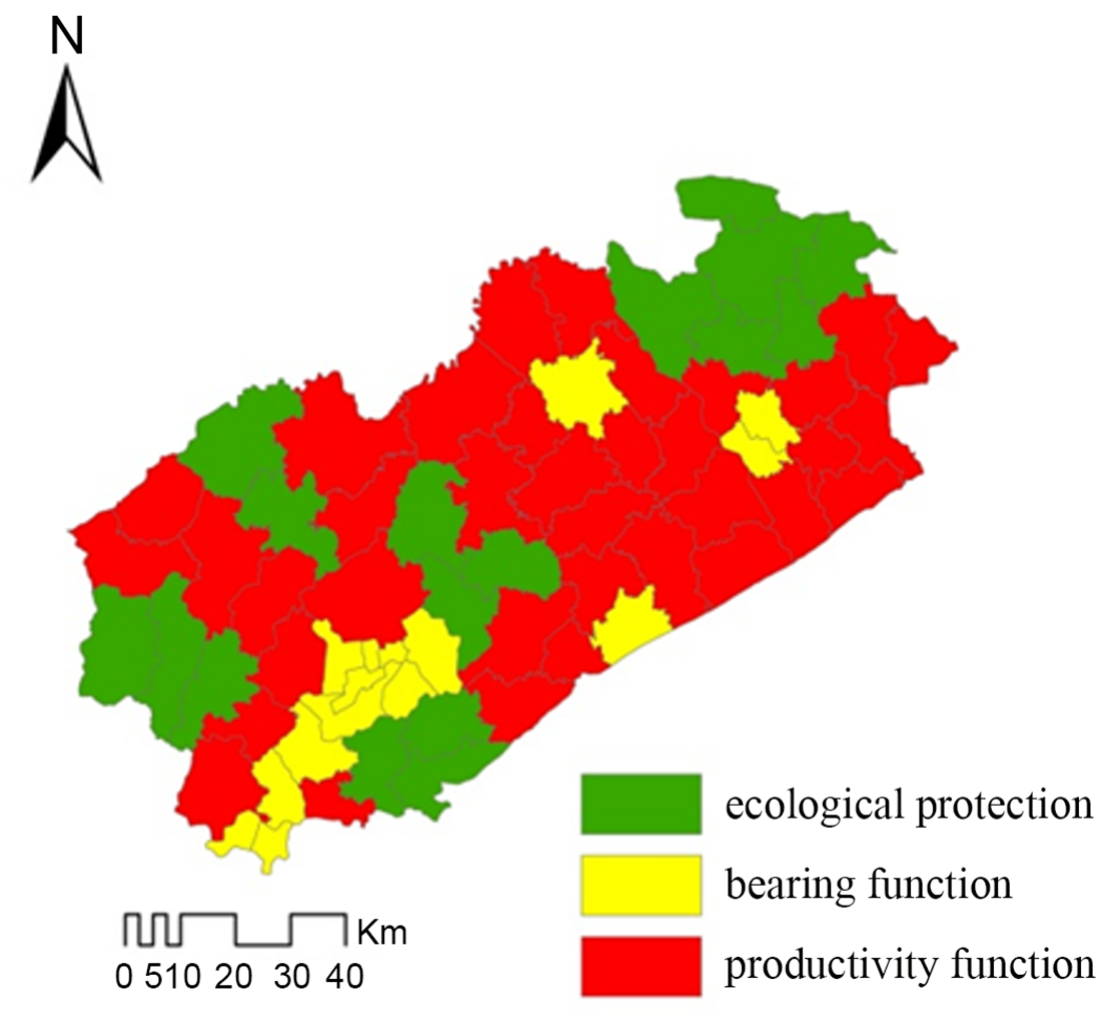
Different soil environmental function areas in Fuxin City.

Synthesizes kinds of situation of Fuxin City, such as the existing land use, soil environmental quality, and the sustainable development in ecology, economy and society, the soil environmental function areas were analyzed and classified in detail (Fig 4). Generally, there are three major environmental functions of soil including productivity function, bearing function and ecological protection function. Fig 4 showed that ecological protection function zone distribution in northeastern and protectionzone and some national scenic spot. Meanwhile, the bearing function zone also distribution in urban area and mining area, most of productivity function zone distribute in farm land.

Industrial transformation plan of Fuxin need to fully consider the quality and function of soil environment. In the ecological protection function area, we must put in practice a strict management system and measures to protect arable land, forest and grasslands. All industrial production and mining activity should be strictly prohibited. In the productivity function area, although the soil was polluted slightly by the heavy metals, the agricultural production can continue with some improvement measures to protect the safety of agriculture products, such as reducing fertilizer and pesticide use, screening of hyperaccumulators or low-accumulation plants[30], and adding soil amendments. In the bearing function area, current situation of soil environment may not suitable for agricultural production directly. The polluted soil should be used after remediation. And it is suggested that this area is best to build some artificial landscape, such as the coal geologicalpark for the urban residents.

## Conclusions

The industrial transformation of resource-exhausted city has become the most talked topic of whole world. As the most representative coal resource-based city of China, Fuxin became the first pilot city of economic transition. Modern agriculture production is one of the most development directions. However, the agriculture production has the inseparable relations with the soil environmental quality. The results of this study showed that the concentrations of most of the heavy metals were found to have increased because of human activities such as mining, industrial production and agriculture production. Many hotspots were contaminated with Cd, Cr, Ni, Pb, and Zn, and these would have been caused by intensive mining production, which has been occurring in the sampling area for nearly 50 years. In addition, uncontrolled agricultural production is an pollution source of heavy metals such as Cu, Pb and Zn, which is often overlooked. According to the Nemerow pollution indices, the level of pollution in soils across Fuxin increased in the order As <Zn < Cr <Pb< Cu <Ni < Cd, and approximately 4,429 km^2^ of Fuxin was found to be slightly polluted. The results of this study will be useful for establishing reasonable land use planning guidelines. According to the present situation of land use, soil environmental quality, and the sustainable development in ecology, economy and society in Fuxin city, the soil were classified into three environmental function areas. Proper soil environmental management policy was proposed to decrease the environmental risks in the process of industry transformation.
